# Hemothorax: When Hemosuccus Pancreaticus Takes an Unexpected Detour to the Chest

**DOI:** 10.7759/cureus.81916

**Published:** 2025-04-08

**Authors:** Arunima Das, T. Raghupathy, Rajendran Shanmugasundaram

**Affiliations:** 1 General Surgery, Sree Balaji Medical College and Hospital, Chennai, IND; 2 General Surgery, Bharath Institute of Higher Education and Research, Chennai, IND; 3 Surgical Gastroenterology, Sree Balaji Medical College and Hospital, Chennai, IND

**Keywords:** acute-on-chronic pancreatitis, emergency tracheostomy, endoscopic retrograde cholangiopancreatography (ercp), hemosuccus pancreaticus, hemothorax, retropharyngeal hematoma

## Abstract

This case highlights an unusual presentation of bilateral pleural effusion associated with chronic pancreatitis, emphasizing that although pleural effusions are typically unilateral and left-sided, they can occasionally be bilateral. In acute or chronic pancreatitis, pleural effusions are generally transient and resolve once the underlying condition is appropriately managed. While most effusions are left-sided, they may rarely occur on the right side or present bilaterally, as observed in this patient. These effusions are typically exudative, with elevated pleural fluid amylase as a key diagnostic indicator of pancreatic origin.

A 37-year-old male with a history of chronic alcohol consumption initially presented with evidence of a right-sided pleural effusion. Elevated amylase levels in the pleural fluid strongly suggested a pancreatic etiology. After a week of hospitalization, the patient developed a left-sided pleural effusion, further complicating the diagnosis. This is a rare and severe complication of hemosuccus pancreaticus (HP), which can result from hemorrhage through the ampulla of Vater or the pancreatic duct, often due to the rupture of surrounding structures such as the splenic artery. Although HP is a challenging diagnosis, computed tomography angiography (CTA) remains the gold standard for identifying the bleeding source and confirming the diagnosis.

Pancreatitis should be strongly considered when a pleural effusion exhibits elevated amylase levels. Prompt identification and treatment of the underlying pancreatic condition and management of complications such as HP are crucial for resolving the effusion and improving the patient's prognosis. Early diagnosis and intervention generally lead to a favorable outcome, as these effusions are typically transient and resolve with appropriate treatment of the underlying pancreatic disorder.

## Introduction

Gallstones and chronic alcohol consumption are the most common causes of acute pancreatitis (AP), an inflammatory disease of the pancreas that can occasionally be triggered by various other factors [1]. Smoking has been associated with an increased incidence of pancreatitis, particularly among individuals who consume alcohol. Additionally, pancreatitis is the most frequent adverse effect following endoscopic retrograde cholangiopancreatography (ERCP), occurring in approximately 3.5% of cases.

AP is frequently classified as mild, moderate, or severe based on the presence and duration of organ failure (lasting less than or more than 48 hours), as well as the presence of local complications, including pseudocysts, acute peripancreatic fluid collections, acute necrotic collections, or walled-off necrosis [2]. Diagnosis is confirmed by integrating radiographic, laboratory, and clinical findings. The primary objectives of supportive care include bowel rest, pain management, fluid resuscitation, and, when necessary, nutritional support. Management is typically conservative, although surgical intervention may be considered if complications arise. Around 25% of patients with pancreatitis experience vascular complications, which are linked to high death and morbidity rates [3]. Hemosuccus pancreaticus (HP) is a rare cause of gastrointestinal (GI) bleeding associated with pancreatic disorders [3]. Characterized by bleeding through the ampulla of Vater, it results from blood within the major pancreatic duct. This condition is most commonly caused by the rupture of a visceral aneurysm into the main pancreatic duct, with splenic artery pseudoaneurysm secondary to chronic pancreatitis being the frequent underlying etiology [3-5]. The accessory pancreatic duct, which empties into the minor duodenal papilla, may occasionally be the site of bleeding. While pseudoaneurysms occur in a notable percentage (3.5%-10%) of pancreatitis patients, their rupture is a rare but critically dangerous complication of chronic pancreatitis. This rupture affects only 6%-8% of patients with pseudocysts and represents a very small fraction (<1%) of upper GI bleeds [5]. If left untreated, the mortality rate approximates 90%; however, with appropriate intervention, it decreases to 25%-37% [6].

## Case presentation

A 37-year-old male presented to the emergency department with complaints of shortness of breath for the past three weeks, not associated with orthopnea. He also reported a dry cough, intermittent abdominal pain, and a 15 kg weight loss over the preceding five months. Abdominal examination revealed tenderness with guarding in the epigastric, umbilical, and left hypochondriac regions.

The patient had been diagnosed with AP five months prior and was treated conservatively. He had a 10-year history of alcohol use and a five-year history of smoking. A chest computed tomography (CT) scan revealed bilateral pleural effusions. A pulmonology consultation was obtained, and an intercostal drain (ICD) was inserted on the right side, draining 500 mL of hemorrhagic fluid. Analysis of the fluid demonstrated significantly elevated serum amylase and lipase levels, which gradually decreased over the subsequent two days (hemoglobin [Hb]: 12.5 g/dL, total leukocyte count [TLC]: 14.51 x 10^9^/L, serum amylase: 576 U/L, serum lipase: 447 U/L, ICD fluid amylase: 100 U/L, and ICD fluid lipase: 70 U/L).

**Figure 1:**
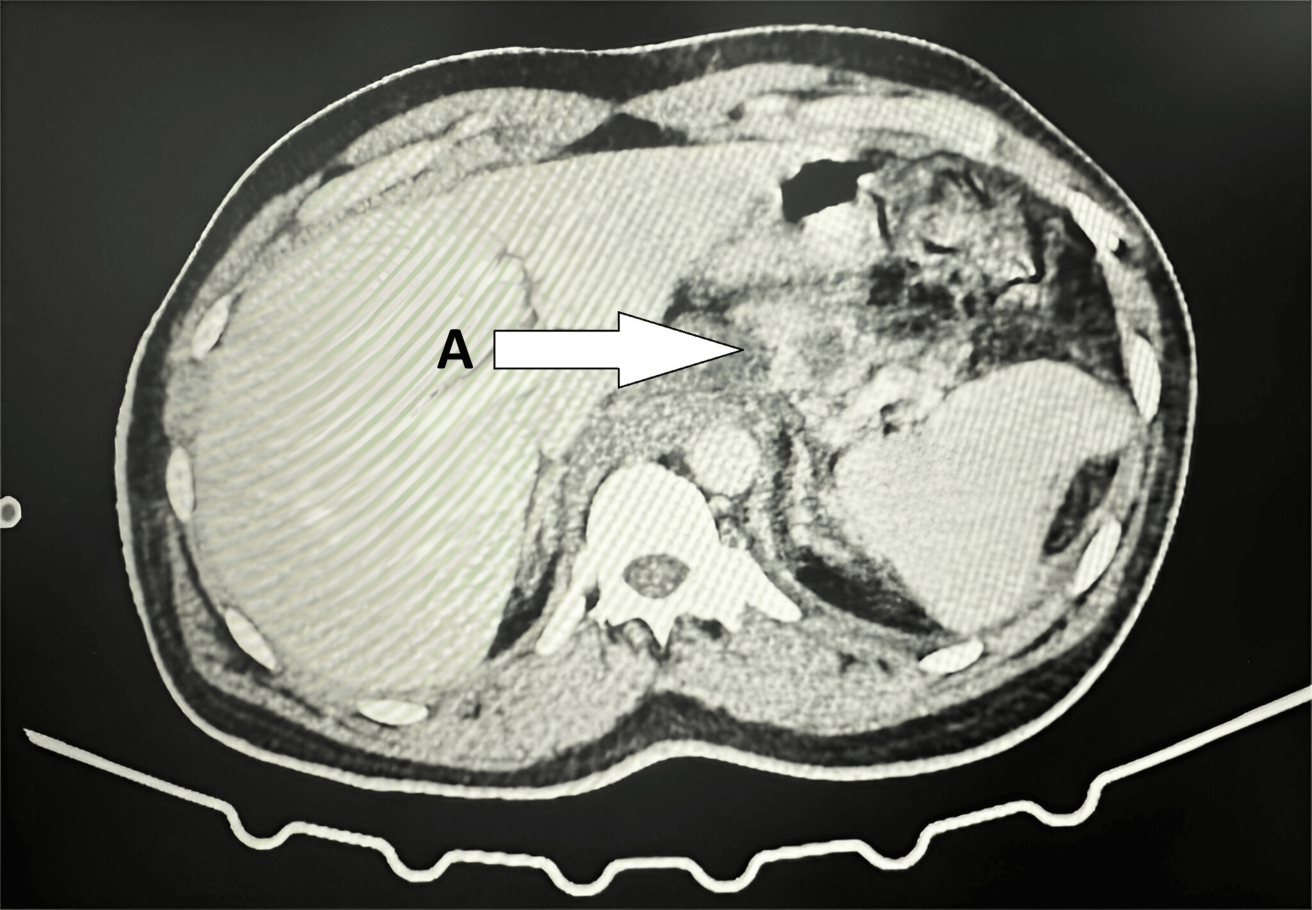
CECT abdomen showing chronic pancreatitis with mediastinal extension of a pseudocyst, peripancreatic fluid collections, and possible ductal disruption in the tail region. A: Chronic pancreatitis with mediastinal extension of a pseudocyst, peripancreatic fluid collections, and possible duct disruption in the tail region. CECT, contrast-enhanced computed tomography

Despite initial intervention, the patient continued to experience tachypnea, tachycardia, and persistent abdominal pain. He was unable to tolerate oral intake and was managed conservatively with nil per os (NPO) status, continuous nasogastric aspiration, intravenous (IV) antibiotics, and IV analgesics. Given the persistent tachycardia, a contrast-enhanced CT (CECT) of the abdomen was performed, revealing chronic pancreatitis with mediastinal extension of a pseudocyst, peripancreatic fluid collections, and possible ductal disruption in the tail region (Figure 1).

**Figure 2:**
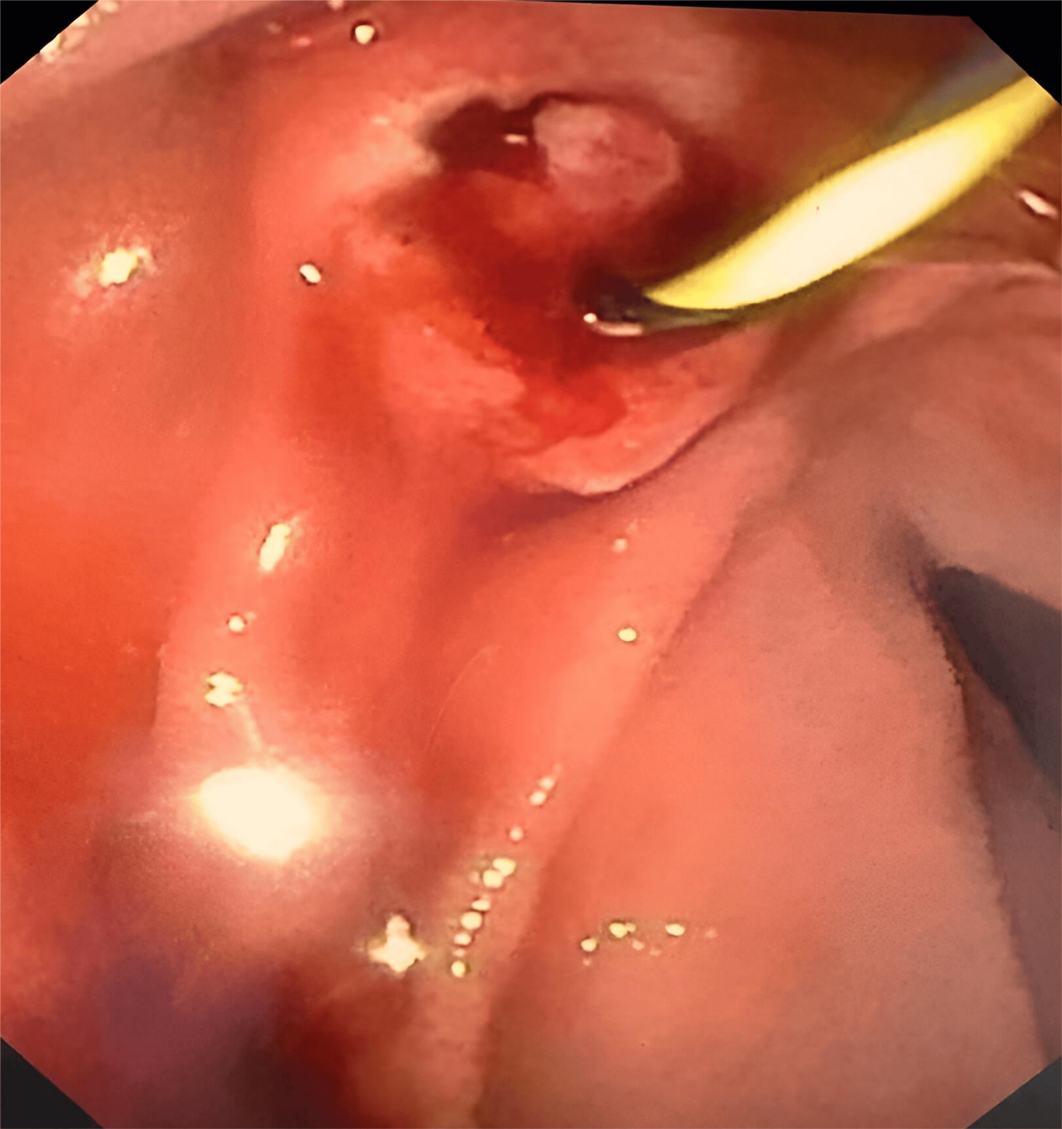
ERCP showing hemosuccus pancreaticus. ERCP, endoscopic retrograde cholangiopancreatography

Serum amylase and lipase levels remained elevated. Consequently, endoscopic retrograde pancreatography (ERP) and stenting were performed to reduce ductal pressure (Figure 2); post-procedure vitals were stabilized.

**Figure 3:**
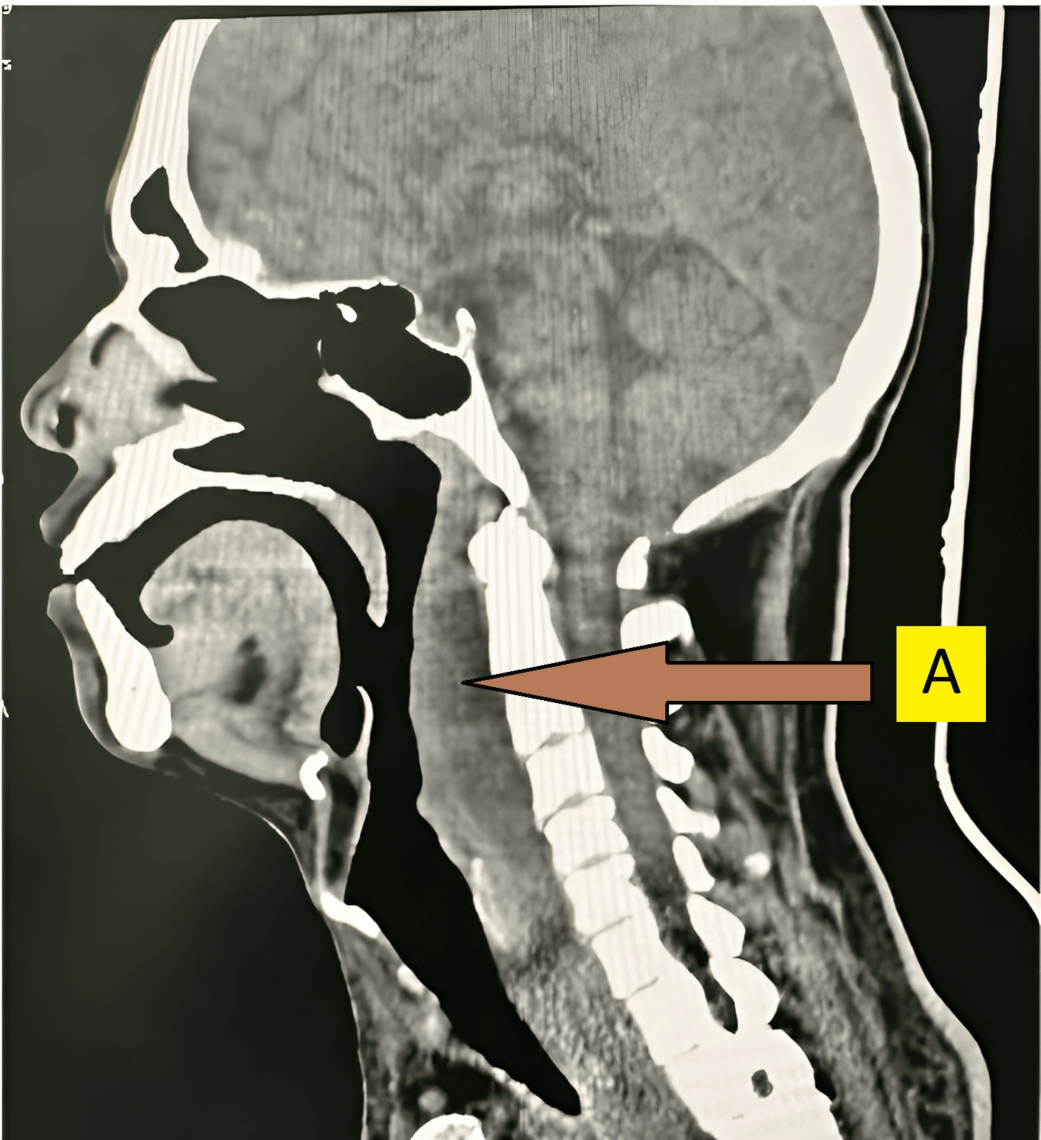
CT of the neck revealed a retropharyngeal abscess. A: Retropharyngeal abscess.

On postoperative day 2, the patient developed neck swelling. Physical examination revealed localized warmth and tenderness but no subcutaneous or surgical emphysema. An otolaryngology (ENT) consultation was obtained, and a retropharyngeal abscess was diagnosed, confirmed by lateral neck X-ray and neck CT scan (Figure 3).

The patient was taken for emergency incision and drainage. Intraoperatively, an infected retropharyngeal hematoma was drained, and a tracheostomy was performed. 

Subsequently, the patient developed oral ulcers. A sputum culture and sensitivity test identified Pseudomonas aeruginosa as sensitive to Tobramycin. Following a pulmonology consultation, nebulized tobramycin was initiated, and the patient’s symptoms improved. Oral intake was gradually resumed. After 15 days of close observation, the tracheostomy was closed.

Following repeated chest radiographs, a pulmonology consultation was obtained, and the ICD was removed. Serial CBC, serum amylase, and lipase monitoring were done within normal limits. The patient was followed up for six months, and no evidence of further complications or recurrence was noted.

## Discussion

HP is an uncommon cause of GI bleeding associated with pancreatic disorders. It occurs when blood from the pancreas enters the GI tract through the ampulla of Vater, usually via the central pancreatic duct. In some cases, bleeding may also occur through the accessory pancreatic duct, draining through the minor duodenal papilla. The bleeding can originate from the pancreas itself, the pancreatic duct, or surrounding blood vessels, such as the splenic or gastroduodenal arteries. HP represents approximately 1 in 1,500 cases of upper GI bleeding and has a notable male predominance (around 7:1). Potential causes of HP include pancreatic inflammation, arterial aneurysms or pancreatic pseudocysts, pancreatic tumors, iatrogenic factors, congenital defects, and trauma [7-9].

Pancreatic inflammation, whether acute, chronic, or hereditary, is found in about 80% of HP cases, often resulting from ductal inflammation that causes the rupture of nearby blood vessel walls. Local irritation from conditions like gallstones or pseudocysts can also lead to vascular inflammation and bleeding. Pseudocysts containing activated enzymes such as elastase may erode adjacent vessels, causing hemorrhage. Arterial aneurysms or pancreatic pseudocyst-associated aneurysms (PSAs), especially in the splenic, gastroduodenal, pancreaticoduodenal, or hepatic arteries, are key contributors to HP [10-12].

A review by Cui et al., which examined cases from 1977 to 2020, found that melena was the most common symptom, occurring in 52% of cases, followed by abdominal pain in 46% [13]. Other symptoms included nausea, vomiting, weight loss, and lower GI bleeding (hematochezia). GI bleeding in HP is typically intermittent, often caused by clot formation in the pancreatic duct that temporarily halts bleeding while abdominal pain persists. Once the clot resolves, re-bleeding can occur, with subsequent pain improvement as intraductal pressure decreases.

Diagnosing HP is challenging owing to its nonspecific and variable clinical presentation. A retrospective study by Yashavanth et al., which reviewed patients with suspected HP over 10 years, reported a median bleeding duration of 10 days before diagnosis. Notably, 40.2% of patients had symptoms persisting for over a month, and 62% demonstrated evidence of visceral artery aneurysms. Upper GI endoscopy detected bleeding in 64.4% of patients, and angiography successfully localized the bleeding source in 94.2% of cases [14].

Serum tests, such as amylase, lipase, or bilirubin levels, are of limited utility in HP. While hyperbilirubinemia may occur due to pancreaticobiliary reflux and amylase and lipase levels may be elevated in pancreatitis, imaging is crucial for diagnosis. Upper GI endoscopy is important for detecting bleeding through the ampulla of Vater, utilizing side-viewing endoscopy, and can help exclude other GI bleeding etiologies. However, its sensitivity for detecting HP ranges from 30% to 65%, likely due to the intermittent nature of the bleeding. ERCP can reveal filling defects in the pancreatic ducts, aiding diagnosis, but it is not commonly used. Doppler ultrasonography may detect aneurysms or pseudocysts, though it is positive in only approximately 38% of cases. Abdominal CT with contrast is frequently used in pancreatic pathology, detecting intraductal blood clots, aneurysms, pseudocysts, or contrast persistence in the arterial phase, and it aids in diagnosing HP in approximately 90% of cases. However, angiography remains the gold standard, accurately identifying the bleeding source, visualizing arterial anatomy, and enabling therapeutic intervention.

Management of HP focuses on stabilizing hemodynamics, providing intravenous hydration and blood transfusions as needed, and controlling the bleeding source. Supportive care alone is insufficient due to the high mortality rate (up to 90%). The treatment approach depends on the patient's hemodynamic status. For stable patients, interventional radiology (IR) procedures such as coil embolization, balloon tamponade, and stent-graft placement have shown immediate success in 79% to 100% of cases, with an overall success rate of 67%. Recurrence rates after IR procedures are approximately 30%, though the cause of recurrence is unclear. One theory suggests that collateral vessels in the adjacent diseased pancreas may contribute to re-bleeding [15].

A pancreatic fistula is an abnormal connection between the pancreas and nearby structures, such as organs, blood vessels, or cavities. These fistulas can be categorized as internal or external, depending on the location of the connection. Although pancreatic fistulas following episodes of pancreatitis have not been extensively researched, case series provide some insights. Internal pancreatic fistulas are more commonly associated with chronic pancreatitis, particularly related to alcohol consumption. These fistulas result from the disruption of the pancreatic duct due to trauma, AP, chronic pancreatitis, or pancreatic resection. Initially, fluid collections may be the only finding, but persistent collections can lead to pseudocyst formation, which may erode nearby structures, causing fistula formation. A 2014 review by Brown et al. found that patients with pancreatic-portal fistulas often had pseudocysts, most of which were located in the head of the pancreas [11].

Clinical symptoms of HP include abdominal pain, abdominal distension (due to pancreatic ascites), nausea, vomiting, shortness of breath (due to pleural effusion), fever, and, in rare cases, life-threatening hemorrhage. Disseminated fat necrosis has been reported, presenting as peripheral subcutaneous fat necrosis, likely caused by pancreatic enzymes entering the systemic circulation. Elevated amylase levels (>6,000 U/L) are consistently observed in these cases. Diagnostic methods such as ultrasound (US) and ERCP are used, although their sensitivity and specificity for detecting pancreatic fistulas are not well-established. Most case series rely on ERCP for both diagnosis and therapeutic intervention, making it the most accurate diagnostic tool. US can detect complex fluid collections in the portal vein, often showing no flow on Doppler imaging. Contrast-enhanced CT (CECT) typically reveals fluid-attenuated areas in the portal vein, with possible collateral periportal vessels and pseudocysts. Magnetic resonance cholangiopancreatography (MRCP) with T2-weighted imaging may show a hyperintense portal vein and fluid signals, occasionally identifying a hyperintense fistulous tract. In cases of portal vein thrombosis, US typically shows absent or decreased flow on Doppler without complex fluid collections, while CT reveals a hypodense portal vein with no contrast enhancement. MRCP with T1-weighted sequences demonstrates an isointense portal vein without fluid signals. Secretin-enhanced MRCP can help further assess pancreatic ducts when diagnostic uncertainty arises.

When the diagnosis of a pancreatic-portal vein fistula is uncertain, percutaneous transhepatic portography (PTP) is a valuable diagnostic tool. PTP accurately maps the portal circulation, determines the fistula's extent, aligns with surgical findings, and enables fluid analysis.

Treatment strategies vary, ranging from conservative to surgical. For stable, minimally symptomatic patients, conservative management can be effective. Endoscopic techniques, such as US-guided cyst drainage and ERCP with pancreatic stent placement, also offer viable options. Surgical interventions, including partial or total pancreatectomy, are reserved for patients with widespread fat necrosis or those who do not respond to or are unsuitable for endoscopic treatments. Due to the acute and variable nature of pancreatic-portal vein fistulas, a standardized treatment protocol is lacking [16].

## Conclusions

The diagnosis and management of HP often require a multidisciplinary approach involving a team of specialists, including primary care physicians, hospitalists, interventional radiologists, emergency medicine physicians, gastroenterologists, hepatologists, and general surgeons. Coordinated care among these specialists is essential to ensure optimal outcomes and deliver high-quality healthcare. Physicians must be well-versed in HP, as effective management demands vigilant monitoring for changes in clinical status, enabling timely intervention.

Hemothorax, a potential complication of HP, underscores the importance of considering uncommon diagnoses when confronted with unusual clinical presentations. It serves as a reminder that anatomical variations can pose significant diagnostic challenges. Achieving an accurate diagnosis of HP requires considerable clinical expertise to prevent prolonged suffering, repeated hospitalizations, and unnecessary blood transfusions. Treatment should be tailored to the patient's clinical condition and guided by the availability and expertise of gastroenterologists and emergency medicine specialists.
